# Contemporary Patient Satisfaction Rates for Three-Piece Inflatable Penile Prostheses

**DOI:** 10.1155/2012/707321

**Published:** 2012-07-26

**Authors:** Raymond M. Bernal, Gerard D. Henry

**Affiliations:** ^1^Division of Urology, Department of Surgery, Duke University Medical Center, Box 3274, Durham, NC 27710, USA; ^2^Regional Urology, 255 Bert Kouns Industrial Loop, Shreveport, LA 71106, USA

## Abstract

Among the many treatments for erectile dysfunction, implantation of a penile prosthesis has been associated with high patient satisfaction rates. Specifically, the placement of a three-piece inflatable penile prosthesis (IPP) confers the highest rates of satisfaction. We reviewed the literature over the past 20 years regarding satisfaction rates for penile prostheses, with a focus on patients who had undergone an initial IPP implantation for erectile dysfunction. In all, 194 articles were reviewed, and of these, nine met inclusion criteria for analysis and data collation. We determined contemporary satisfaction rates to reflect patients' experiences with newer products and surgical approaches. Of importance, we noted that varied metrics were used to determine patient satisfaction, and overall satisfaction could not be precisely determined. Nevertheless, we found that patients in general were quite satisfied with their three-piece IPPs and restoration of sexual function. We also identified reasons for patient dissatisfaction and reviewed the literature to find ways by which satisfaction could be improved. Given the various means by which patient satisfaction was determined, future efforts should include standardized and validated questionnaires.

## 1. Introduction

The placement of a patient-activated inflatable penile prosthesis (IPP) to treat erectile dysfunction has allowed patients to achieve dependable spontaneity for intercourse. As compared with other treatments for erectile dysfunction, including oral medication, transurethral suppositories, injectable medications, and vacuum-assisted devices, patients who have a penile prosthesis have reported the highest satisfaction rates [1–4].

Early satisfaction rates had been determined by physicians' assessments. However, discrepancy has been shown between satisfaction rates determined by physicians and those determined by patients [5]. It is generally thought that patient self-administered questionnaires are more reliable than those administered by a physician. As such, over the past several years, penile prosthesis satisfaction rates have been captured by self-administered surveys. However, relatively few studies have been conducted utilizing validated surveys.

Early satisfaction rates for penile prosthetic implants do not reflect contemporary device improvements or surgical technique. Indeed, over the years, device manufacturers have modified their penile prostheses to improve device satisfaction and longevity rates. Having evolved from malleable and two-piece penile implants, the three-piece inflatable penile prosthesis reflects the most modern implantable device. The highest patient-reported rates of satisfaction have been associated with the three-piece IPP [6]. The most commonly implanted multicomponent prostheses today are manufactured by two companies: American Medical Systems (AMS, Minneapolis, MN) and Coloplast (Copenhagen, Denmark). We review the literature specifically pertaining to satisfaction rates after three-piece IPP implantation over the past 20 years. Moreover, we investigate if satisfaction rates changed over time, based upon the self-report instruments, new device modifications, improvements in surgical techniques, and realization that preoperative counseling is important.

## 2. Methods

A Pubmed literature search was conducted using the search terms “penile prosthesis” and “satisfaction”, “quality of life”, or “outcomes”. A total of 194 abstracts were identified that noted satisfaction rates associated with penile prostheses. Literature that reported patient satisfaction for initial “virgin” recipients of three-piece IPPs were identified and analyzed.

Articles published more than 20 years ago were excluded, as were those written in non-English language. Articles that did not specify the type of prosthesis implanted, as well as those that grouped self-contained prostheses into their satisfaction analyses, were not considered. Series with less than 30 patients were not included. Articles that included concurrent operations that may have influenced satisfaction, such as release of the dorsal penile suspensory ligament, were also excluded.

## 3. Results

Nine articles that were published over the past 20 years met criteria for review, such that we might address patient satisfaction rates after initial three-piece IPP placement specifically (see Table 1). All questionnaires used in these studies were self-administered, but only one article determined satisfaction by means of a validated questionnaire.

Bettochi and colleagues from Italy collected information from 79 patients and their partners after implantation of an AMS CX 700 prosthesis [7]. This was a single-surgeon and single-center study for implantations performed from 2004 to 2008. To help eliminate bias, a nine-point questionnaire was administered by telephone by a neutral interviewer. Among the 79 patients, 97% noted frequent use of the prosthesis. Those who did not use it frequently were no longer sexually active. At the time of interview, 85% of patients and 98% of partners reported no problems with the prosthesis. And 79% of patients and 82% of their partners indicated that penile prosthesis implantation led to satisfying improvements in their sexual life, with an additional 13% of patients reporting slight improvements. Of the 8% who were unsatisfied, reported reasons included insufficient rigidity and penile length for normal intercourse. On a note of interest, despite dissatisfaction with the device, four of these six patients said they would still recommend surgery, because they observed an improvement in couple relationship satisfaction. Overall, 97% of patients would suggest this treatment to a friend or relative with erectile dysfunction [7].

Another European study from Natali and colleagues sought to quantify satisfaction with AMS penile implants from 253 consecutive patients at three European centers in Italy and Germany [8]. Satisfaction data were determined by using a self-administered modified EDITS (erectile dysfunction inventory of treatment satisfaction) questionnaire, which is a validated instrument. It should be noted that of the 253 consecutive patients, 53 were lost to followup, and of the remaining 200, 40 had postoperative complications. These patients were excluded, and of the 160 patients without major postoperative complications, 115 returned the mailed EDITS questionnaire, 33 of which had a three-piece IPP implanted. With these limitations in mind, and acknowledging that postoperative complications are lower in the United States, overall patient satisfaction for the AMS 700CX in those surveyed was 97%. Specifically, 67% were very satisfied, 30% were somewhat satisfied, 3% noted neither satisfaction nor dissatisfaction, and 0% reported dissatisfaction; 91% felt that expectations were at least considerably met and 97% were likely to continue using their prosthesis. Furthermore, 91% felt confident in having sex and that their partner was satisfied [8].

Brinkman and colleagues identified 1298 patients who received various virgin three-piece IPPs and randomly sampled 330 patients [9]. These patients' surgeries were performed by the same surgical team at one hospital between 1992 and 1998. Three types of prostheses were implanted, including the AMS 700 Series, Mentor Alpha 1, and Mentor Alpha NB. Patients were interviewed by telephone using a survey developed by the authors. In all, 248 patients responded to the question: “How satisfied are you with the prosthesis?” Of these, 17 had AMS implants and 231 had Mentor implants. The overall satisfaction rate was 69%, and there were no statistically significant differences among satisfaction rates based on implant type. An additional 11% were neither satisfied nor dissatisfied, 80% would have the implant surgery repeated, and 84% would recommend surgery to others [9].

Carson and colleagues performed a multicenter study regarding the AMS 700CX implantation in men with a mean followup of four years [10]. Seven “frequent implanter” surgeons contributed to this study of patients from 1987 to 1996, which included a telephone survey of 207 men by a neutral interviewer. At the time of the interview, 178 men still had their implant, including 89.7% with a regular sexual partner. Of these, 87.1% were able to generate erections sufficient for intercourse and 79% used it at least twice monthly. In addition, 76.2% of surveyed men were satisfied or highly satisfied with device function, 86.5% would undergo the penile prosthesis implantation again, and 88.2% would recommend an implant to a relative or friend [10].

Montorsi and colleagues studied AMS three-piece IPPs in 200 patients and 120 partners at a mean of 59 months (range 6–130) postsurgery, and they were extensively questioned about function of the device and its impact on the couple's sexual life [11]. At the time of inquiry, 185 patients (92.5%) were still engaging in sexual intercourse with a mean frequency of 1.7 times per week. Patients and partners reported prosthetic erections as excellent or satisfactory in 98% of cases and 83% of cases, respectively. Postoperative sexual activity was considered excellent or satisfactory in 92% of patients and 96% of partners, respectively [11].

Holloway and Farah looked at 145 patients who underwent implantation of an AMS700 Ultrex penile prosthesis with a mean followup of 42 months [12]. Patient responses to the authors' questionnaire showed that 85% were satisfied. Overall, 85% had durable and reliable implant function and 86% had a sustained level of satisfaction with the implant. Overall satisfaction with the device reported by the sexual partner was 76% [12].

Goldstein and colleagues conducted a two-phase study based on 434 implantations from seven surgeons from March to October 1993 [13]. Results from implantation of Mentor Alpha-1 three-piece IPPs were studied, with 234 responding to a mailed questionnaire. Satisfaction responses of 80% or greater were noted with regard to intercourse ability (83%) and confidence (80%), as well as device rigidity (84%) and function (84%). Among the respondents, 89% of patients reported fulfilled expectations with the Alpha-1 prosthesis as treatment for their erectile dysfunction [13].

Garber evaluated a series of 50 men implanted with a Mentor Alpha 1 at a mean followup of 15 months [14]. In this study, 98% of the patients and 96% of their partners were satisfied with the device, and 94% and 96% thought the device was easy to inflate and deflate, respectively. All were satisfied with the girth and rigidity, but only 92% were satisfied with the length; yet 98% said they would undergo the procedure again and would recommend this implant to other patients [14].

Goldstein and colleagues followed 112 patients after implantation of a Mentor Alpha-1 IPP [15]. Surgeries were performed by 12 implanters with varied surgical backgrounds. At a mean followup of 27 months, 96 of 112 surveys were returned and analyzed. Among these patients, 82% stated that the device fulfilled expectations as a treatment for impotence, and 83% had improved sexual intercourse by 8 weeks after implantation. Patient satisfaction was computed on a scale of 12 equally weighted interrelated variables. Among the patients, 77% recorded nine or more cumulative satisfaction points [15].

## 4. Discussion

Only one of the nine studies used a validated patient self-reported questionnaire to assess satisfaction rates. The EDITS questionnaire was first validated in 1999 as an instrument by which patients' and partners' satisfaction with treatments for erectile dysfunction could be assessed. This was done in acknowledgement of the subjective nature of patient satisfaction and that it encompassed more than treatment efficacy [16]. Satisfaction rates in articles that met inclusion criteria were self-reported by patients, but the assessment of patient satisfaction across all studies was not standardized. Overall satisfaction rates, satisfaction with the device, satisfaction with sexual relationship postprocedure, willingness to undergo the procedure again, and willingness to suggest implantation to family and friends as a treatment option were different metrics by which satisfaction could be suggested. The Carson et al. and Brinkmann et al. studies obtained all these data and more with excellent satisfaction rates across multiple questions [9, 10]. Because authors' definitions of patient satisfaction were not consistent across studies, as some used graded scales to assess satisfaction while others used single questions to assess satisfaction, total overall satisfaction in this paper cannot be determined. Significant additional limitations to these studies include sampling bias, loss of patients to followup, the varied experience of the prosthetic surgeons, and relatively small numbers of patients surveyed. Nevertheless, all of the studies that met inclusion criteria showed that patients are highly satisfied with implantation as a method of treatment for their erectile dysfunction.

Satisfaction can be affected by many variables. Partners' attitudes may play a role [17] and patient expectations can have a great impact [18]. Poor outcomes requiring explantation and secondary procedures affected responses. For patients who reported being dissatisfied with their IPPs, noted complaints included loss of perceived length [11, 19], poor glandular engorgement [11], report of unnaturalness by partner [20], pain [21], difficulty with pump inflation and deflation [22], partner feelings of dissatisfaction [13], and complications requiring device removal, such as infection or erosion [23]. However, it is not surprising that there were no statistical differences between the two companies' IPP products, as shown in the Brinkmann et al. study [9].

Modifications of the devices and surgical techniques, as well as medication and behavioral therapies, have been developed to address some of these complaints. Prosthetics companies have made improvements in their pump hydraulic systems to allow easier handling and deflation. Modifications in pump and coating characteristics of the cylinders have increased long-term mechanical reliability [24]. The tips of the prostheses have been made softer for a more natural feel during intercourse. Surgical techniques have been described to maximize penile length. New length measurement techniques (NLMT) used to allow for use of larger cylinders have been described [25]. A ventral phalloplasty technique may increase patients' perception of phallic length [26].

Methods by which satisfaction can be improved after implantation have been described. For patients who complain of cold glans syndrome, oral PDE-5 inhibitors, as well as intraurethral alprostadil suppositories, can be utilized to help with glans engorgement [27]. The use of a vacuum erection device has been suggested to help augment rigidity and engorgement in patients unfit for or unwilling to undergo implantation surgery [28]. Behavioral strategies, including the use of foreplay and different sexual positions, can be used to make intercourse more enjoyable. Sex therapy and counseling can also augment satisfaction after implantation [29]. As the Holloway and Farah study shows, usually the patient satisfaction rates are higher than the partner satisfaction rates; thus, sex therapy and counseling could possibly help partner rates even more than patient satisfaction rates [12].

Of the 8% who were unsatisfied in the Bettochi et al. paper, the main reasons given were insufficient rigidity and penile length, which some experts feel could be addressed by better instructions on using the pump given to the patient and use of the NLMT, respectively [7, 25]. Moreover, the same could be said for the Garber study where 8% were dissatisfied with penile length. Again, perhaps applying NLMT, which consists of surgeons choosing longer instead of shorter lengths for cylinder and rear tip extenders, may improve satisfaction rates [14, 25]. Natali et al. had 40 or 200 patients with post-operative complications, with improved devices and better surgical techniques that complication rate could be lowered significantly [8]. Therefore, despite already high rates of patient satisfaction rates with IPPs, there are several means by which to pursue even higher rates in the future.

## 5. Conclusion

Nine studies over the past 20 years show that patients with erectile dysfunction who undergo three-piece IPP placement report high satisfaction rates. Patient satisfaction is clearly affected by many parameters, including patient expectations, partner attitudes, and the presence or absence of surgical complications and premature device failures. By choosing the appropriate candidate for surgery and with careful attention to surgical technique, infection prophylaxis, and post-operative counseling, satisfaction rates can be optimized. Unfortunately, given the variability by which satisfaction rates were assessed in these articles, precise enumeration of overall patient satisfaction could not be determined. However, it is evident that three-piece IPP recipients are generally satisfied with their device and restoration of erectile function. Future efforts to determine patient satisfaction rates should include standardized and validated questionnaires to assess treatment outcomes.

## Figures and Tables

**Table 1:** Summary of studies.

Author	Number of patients	Time of implant placement	Mean followup (yrs)	Satisfaction metrics	%
Bettochi et al. [7]	79	2004–2008	2.8	Frequent use of penile prosthesis	97
				Improvement in sex	92
				Would recommend surgery to others	97

Natali et al. [8]	33	1990–2004	5	Patient satisfaction	97
				Considerably met expectations	91
				Likelihood of continued use	97
				Lack of difficulty in use	91
				Confidence in having sex	91
				Assessment of partner satisfaction	91
				Feels partner wants continued use	97
				Same or improved hardness before ED	100

Brinkman et al. [9]	248	1992–1998	range: 2–8 years	Satisfied	69
				Satisfied or ambivalent	80
				Would have surgery again	80
				Would recommend surgery	84

Carson et al. [10]	207	1987–1996	7.2	Satisfied 4-5 on 5-point scale	76
				Erection suitable for sex	87
				Use at least twice monthly	79
				Recommend to friend/relative	88

Montorsi et al. [11]	200	1986–1997	4.9	Still having sex	93
				Satisfactory erections	98
				Satisfactory sexual activity	92

Holloway and Farah [12]	145	1990–1994	3.5	Overall satisfaction	85
				Sustained satisfaction	86
				Partner satisfaction	76

Goldstein et al. [13]	234	1989–1993	1.9	Fulfilled expectations	89
				Ability to have intercourse	83
				Confidence with intercourse	80
				Device rigidity	84
				Device function	84
				Recommend surgery	86

Garber [14]	50	Pre-1994	1.25	Satisfied with device	98
				Partner satisfied with device	96
				Would undergo procedure again	98
				Would recommend surgery	98

Goldstein et al. [15]	96	1989–1991	2.25	Fulfilled expectations	82
				Satisfaction 9 or better on 12-pt scale	77
